# Characterization of the gut microbiome in Alzheimer disease and mild cognitive impairment among older adults in Uganda: A case–control study

**DOI:** 10.1097/MD.0000000000042100

**Published:** 2025-04-18

**Authors:** Kamada Lwere, Haruna Muwonge, Hakim Sendagire, Martha Sajatovic, Scott M. Williams, Joy Louise Gumukiriza-Onoria, Denis Buwembo, William Buwembo, Rita Nassanga, Rheem Nakimbugwe, Aisha Nazziwa, Ian Guyton Munabi, Noeline Nakasujja, Mark Kaddumukasa

**Affiliations:** a Department of Microbiology, School of Biomedical Sciences, College of Health Sciences, Makerere University, Kampala, Uganda; b Department of Microbiology, Faculty of Health Sciences, Soroti University, Soroti, Uganda; c Habib Medical School, Faculty of Health Sciences, Islamic University in Uganda, Kampala, Uganda; d Department of Physiology, School of Biomedical Sciences, College of Health Sciences, Makerere University, Kampala, Uganda; e Neurological and Behavioral Outcomes Center, University Hospitals Cleveland Medical Center, Case Western Reserve University School of Medicine, Cleveland, OH; f Department of Population and Quantitative Health Sciences, School of Medicine, Case Western Reserve University, Cleveland, OH; g Department of Psychiatry, School of Medicine, College of Health Sciences, Makerere University, Kampala, Uganda; h School of Public Health, College of Health Sciences, Makerere University, Kampala, Uganda; i Department of Anatomy, School of Biomedical Sciences, College of Health Sciences, Makerere University, Kampala, Uganda; j Department of Radiology, School of Medicine, College of Health Sciences, Makerere University, Kampala, Uganda; k Department of Medicine, School of Medicine, College of Health Sciences, Makerere University, Kampala, Uganda.

**Keywords:** Alzheimer disease, gut microbiome, mild cognitive impairment

## Abstract

Alzheimer disease (AD) is associated with significant shifts in the gut microbiome and is characterized by reduced microbial diversity and changes in the abundance of specific taxa. These alterations can disrupt the gut-brain axis, leading to increased intestinal permeability (“leaky gut”), systemic inflammation, and oxidative stress. Such microbial changes are thought to contribute to neurodegenerative changes, as observed in AD and cognitive decline, thus emphasizing the role of the microbiome in aging-related neurological health. Our study in urban and rural population in Uganda recruited 104 participants aged 60 years and older, categorized into AD, mild cognitive impairment (MCI), and control groups based on Montreal Cognitive Assessment (MoCA) scores and ICD-11/DSM-V criteria. DNA was extracted from fecal samples using a QIAamp kit and polymerase chain reaction (PCR) products were sequenced using Nanopore. We used diversity indices, principal coordinate analysis (PCoA), permutational multivariate analysis of variance (PERMANOVA), and linear discriminant analysis effect size (LefSe) to identify significant microbial differences among groups. Gut microbiome diversity, as measured by the Chao1 and Shannon indices, was significantly reduced in patients with AD. The AD group had the lowest diversity compared to that of the control group (*P* < .05). PCoA showed distinct microbial shifts between patients with AD and controls, with MCI showing an intermediate profile. Genera such as *Novosphingobium and Staphylococcus* were more prevalent in the controls, whereas *Hafnia-Obesumbacterium and Dickeya* were more common in AD. Age-related changes included increases in *Exiguobacterium and Carnobacterium* and decreases in *Acinetobacter and Klebsiella*. Distinct microbial profiles were identified in the AD, MCI, and control groups, suggesting potential microbiome markers of cognitive impairment in the Ugandan population.

## 1. Introduction

Alzheimer disease (AD) is a progressive neurodegenerative disorder characterized by memory loss, cognitive decline, and behavioral changes.^[1]^ Pathologically, AD is associated with the accumulation of amyloid-beta plaques and neurofibrillary tangles, leading to significant cognitive and functional impairments.^[1]^ Mild cognitive impairment (MCI) often precedes AD, representing a continuum of cognitive decline.^[2,3]^

Globally, approximately 55 million individuals are affected by dementia, with projections indicating an increase to 152 million by 2050.^[4]^ Approximately 60% of dementia cases occur in low- and middle-income countries (LMICs) where healthcare systems experience significant resource limitations. In sub-Saharan Africa, including Uganda, the prevalence of dementia among individuals aged ≥ 60 years is estimated to be 7.2%, with some studies reporting up to 20% in this age group.^[5,6]^ Notwithstanding these concerning statistics, the etiological mechanisms of AD in African populations remain inadequately elucidated, primarily due to the insufficient representation of these populations in global research initiatives.

Emerging evidence implicates the gut microbiome in the etiology of AD and MCI. Individuals with AD exhibit significant alterations in gut microbiota composition compared to cognitively healthy controls, characterized by reduced microbial diversity and shifts in taxa abundance.^[7,8]^ Pro-inflammatory genera, such as *Escherichia/Shigella*, are elevated, while anti-inflammatory genera, such as *Faecalibacterium* and *Eubacterium*, are depleted.^[9]^ These alterations in the microbial community are hypothesized to modulate neuroinflammation and oxidative stress, which constitute 2 principal mechanisms in AD pathogenesis.^[10,11]^

Gut dysbiosis can induce systemic inflammation by increasing the production of pro-inflammatory cytokines such as interleukin-6 and tumor necrosis factor-alpha.^[12,13]^ These cytokines traverse the blood-brain barrier, activate microglia, and facilitate the accumulation of Aβ plaques and tau tangles.^[14,15]^ Oxidative stress, induced by microbial metabolites, further exacerbates neuronal damage through lipid peroxidation, protein oxidation, and DNA damage.^[16,17]^

Cytokines activate microglia, the resident immune cells in the brain. Activation of microglia leads to chronic neuroinflammation, which contributes to neurodegenerative processes by facilitating the accumulation of amyloid-beta plaques and tau tangles, both of which are pathological hallmarks of AD. The accumulation of these proteins disrupts neuronal communication, ultimately contributing to cognitive decline.^[18]^ Furthermore, oxidative stress induces lipid peroxidation, protein oxidation, and DNA damage in neurons, thereby compromising their integrity and function and disrupting essential signaling pathways involved in learning and memory processes.^[19]^ The gut-brain axis, a bidirectional communication network between the gastrointestinal tract and the central nervous system, serves as a crucial pathway through which the gut microbiota influence brain function, with the vagus nerve functioning as a primary conduit for this communication. In the context of gut dysbiosis, alterations in microbial composition can lead to changes in the production of neuroactive compounds, such as short-chain fatty acids.While short-chain fatty acids, exemplified by butyrate, generally exhibit anti-inflammatory properties, dysbiosis reduces their production, thereby diminishing their neuroprotective effects and further contributing to neuroinflammation.^[20]^ Alterations in vagal signaling resulting from dysbiosis may lead to aberrant brain signaling, potentially exacerbating neuroinflammation and cognitive impairment.^[21]^ Furthermore, dysbiosis may result in a reduction of beneficial metabolites such as tryptophan, which subsequently diminishes the synthesis of serotonin—a neurotransmitter essential for mood regulation—thereby contributing to depressive symptoms, which frequently occur comorbidly with cognitive decline.

While substantial evidence has established a link between gut dysbiosis and AD in Western populations, limited knowledge exists regarding these interactions in African populations, where distinct genetic and environmental factors may result in different microbial signatures and disease mechanisms. This study addresses this knowledge gap by investigating the role of the gut microbiome in AD and MCI within a Ugandan cohort. Specifically, the aim was to characterize the gut microbiome profiles of patients with AD and MCI in comparison to those of healthy controls.

This study presents a novel perspective by focusing on a population underrepresented in AD research. The findings of this study may inform the development of targeted interventions, such as dietary modifications and probiotics, tailored to the specific needs of the African population. Moreover, the data generated in this investigation may provide a valuable foundation for future computational models that can elucidate the complex dynamics between the gut microbiome and neurodegeneration.^[22,23]^

By addressing critical knowledge gaps and providing culturally appropriate strategies for mitigating cognitive decline, this study contributes to the regional and global efforts to combat AD. This research represents a significant advancement in understanding and addressing the increasing burden of AD in sub-Saharan Africa.

## 2. Methods

### 2.1. Study area

The study was conducted in Wakiso District, Uganda, and in the Nansana, Busukuma, and Nakwero communities. This region is characterized by a diverse demographic composition spanning urban, suburban, and rural areas, with an approximate population of 2 million.^[24]^ A community-based door-to-door recruitment strategy was used to identify the elderly individuals with AD. The initial selection was based on health history reviews during the pre-recruitment visits. Subsequently, cognitive assessments and clinical examinations were conducted to confirm the participant eligibility.

### 2.2. Study population

The participants were individuals aged 60 years and older. Recruitment was facilitated by community leaders, such as Local Councilors and Village Health Teams. The study population comprised 77 individuals diagnosed with AD, 14 with MCI, and 13 cognitively healthy controls. The cases were selected from a prior dementia study cohort of 500 elderly individuals from Nansana and Busukuma representing both urban and rural areas.

### 2.3. Clinical assessment and diagnoses

Cognitive screening was performed by trained interviewers using the Montreal Cognitive Assessmen (MoCA). The MoCA cutoff values were set at ≥ 25 for normal controls, 18 to 25 for MCI, and ≤ 17 for dementia.^[25,26]^ Clinical evaluation was conducted by 2 psychiatrists who adhered to the ICD-11 and DSM-5 criteria. During this evaluation, the team used a systematic approach to rule out other forms of dementia such as vascular dementia, frontotemporal dementia, and Lewy body dementia to ensure that the focus remained on AD. Participants were classified as normal, MCI, or AD based on a consensus clinical diagnosis by psychiatrists and a neuropsychologist.^[27,28]^ Cognitively healthy controls were mostly participants’ spouses, to ensure similarity in diet and environment.

Structured questionnaires were used to collect demographic and clinical information including age, education, medical history, lifestyle factors, and cognitive health status. The questionnaires were administered through face-to-face interviews conducted by trained research assistants. The primary language of the questionnaire was Luganda, which is widely used in the study region. However, the English versions were available for participants who were fluent in English. The questionnaire was pretested with a small group of older adults to ensure clarity, cultural appropriateness, and ease of understanding. Feedback from this pretest led to minor revisions to improve comprehension and consistency. The exclusion criteria for both cases and controls were major psychiatric disorders and significant neurological conditions in addition to dementia. Participants with severe systemic diseases such as chronic kidney disease, sepsis, heart failure, diabetes, and recent antibiotic use within 6 weeks were also excluded.

### 2.4. Sample size estimates

We used the G*Power software (version 3.1.9.7) to estimate the required sample size per group.

The formula used to estimate the sample size per group is as follows:


$$\begin{array}{l} n=\left({\left(Z_{1-\alpha /2}+Z_{1-\beta }\;to\;show\;how\right)}^{2}*\left(p1\left(1-p1\right)+p2\left(1-p2\right)\right)\right)/{\left(p1-p2\right)}^{2}\; \end{array}$$

where:

n: Sample size per group

$Z_{1-\alpha /2}$: *Z*-score corresponding to the desired significance level (e.g., 1.96, 95% confidence level).

$Z_{1-\beta }$: *Z*-score corresponding to the desired power (e.g., 0.84 for 80% power).

p1: Proportion in the first group (AD group).

p2: Proportion in the second group (Control group).

(p1–p2): Expected difference in proportions (effect size).

#### 2.4.1. Application

Expected difference in microbial diversity or taxa abundance: We assumed an expected difference of 0.5 between the AD group or MCI and the control group, representing a medium effect size.

#### 2.4.2. Proportions (p1 and p2)

p1 = 0.7: Proportion of interest in the AD group.

p2 = 0.2: Proportion of interest in the control group.

Significance level ($Z_{1-\alpha /2}$): We used a *Z*-score of 1.96 for a 95% confidence level, meaning that we allowed 5% Types I error rate.

Power ($Z_{1-\beta }$): A *Z*-score of 0.84 corresponds to 80% power, meaning we want to detect a true difference 80% of the time, allowing a 20% chance of a Type II error.

#### 2.4.3. Substituting into the formula


$$\begin{array}{l} n=\left({\left(1.96+0.84\right)}^{2}*\left(0.7\left(1-0.7\right)+0.2\left(1-0.2\right)\right)\right)/{\left(0.7-0.2\right)}^{2}\; \end{array}$$

#### 2.4.4. Breaking down the calculation step by step

1. Sum of *Z*-scores: 1.96 + 0.84 = 2.8
2. Square of sum of *Z*-scores: 2.8^2 = 7.84
3. Calculate proportions:

- p1(1 - p1) = 0.7 × 0.3 = 0.21
- p2(1 - p2) = 0.2 × 0.8 = 0.16
- p1(1 - p1) + p2(1 - p2) = 0.21 + 0.16 = 0.37

4. Difference in proportions squared: (0.7–0.2)^2^ = 0.5^2^ = 0.25
5. Final calculation:

n = (7.84 × 0.37)/ 0.25 ≈ 11.6

Rounding up n ≈ 12 per group was required.

#### 2.4.5. G power calculation

Using G*Power version 3.1.9.7, we selected the following parameters to calculate sample size:

Test family: *Z*-tests

Statistical test: proportions: difference between 2 independent proportions (2 groups)

Tail(s): Two-tailed

Effect size: 0.5

Significance level (α): 0.05

Power (1 − β): 0.80

The G*Power output confirmed that a sample size of 12 participants per group was sufficient to detect a medium effect size of 0.5 with 80% power at a significance level of 0.05.

#### 2.4.6. Justification

Thus, a sample size of 12 participants per group was calculated using G*Power software and the given formula, ensuring that the study was adequately powered to detect significant differences in microbial diversity or taxa abundance between the AD, MCI, and control groups at the specified confidence level and power.

### 2.5. Efforts to minimize bias

To minimize potential bias, this study recruited diverse participants from urban and rural Uganda, using local leaders to ensure representation and mitigate selection bias. Standardized diagnostic criteria (MoCA, ICD-11, and DSM-5) and blinding were used to maintain consistency. Controls, often spouses, ensure a similar environment. The exclusion of psychiatric or systemic diseases and recent antibiotic use reduced confounding effects on the gut microbiome. Rigorous sample handling, standardized lab protocols, advanced statistical analyses, principal coordinates analysis (PCoA), permutational multivariate analysis of variance (PERMANOVA), and linear discriminant analysis effect size (LEfSe) helped account for confounders such as age and body mass index (BMI). Multiple diversity indices and validation methods confirmed these findings, enhancing the reliability and generalizability of the study in exploring cognitive impairment.

### 2.6. Ethical approval

The study protocol was reviewed and approved by the School of Biomedical Sciences Research and Ethics Committee of Makerere University (approval number SBS-2022-256) and Uganda National Council of Science and Technology (approval number HS2930ES).

### 2.7. Fecal sample collection, DNA isolation, and sequencing

Rectal swabs were collected by a trained laboratory technician at the participants’ homes, using sterile techniques to prevent contamination. The swab tip was gently inserted up to 3 cm into the rectum and rotated for approximately 5 seconds to ensure adequate sample collection. A depth of 3 cm was selected based on established guidelines for accessing fecal material near the rectal mucosa, which is critical for accurately representing gut microbial composition. A rotation time of 5 seconds was chosen to ensure sufficient contact with the rectal wall to collect a representative sample, while minimizing participant discomfort. After careful withdrawal, the swabs were immediately placed in transport tubes and transported to the laboratory on ice. Transporting samples on ice is essential for preserving microbial integrity as it prevents the overgrowth of bacteria or DNA degradation. Upon arrival, the samples were stored in 2 mL of ASL buffer at −20°C to stabilize the DNA and prevent microbial changes. Further laboratory analyses were conducted within 2 weeks to ensure sample freshness and to minimize any loss in microbial diversity or DNA quality.

### 2.8. DNA isolation

DNA was extracted from rectal swab samples using a modified QIAamp DNA Stool Mini Kit (Qiagen, Hilden, Germany). Upon thawing, DNA was isolated from the swabs using the manufacturer protocol (QIAamp Fast DNA Stool Mini Kit [Qiagen]).

### 2.9. Polymerase chain reaction

PCR was performed to amplify the 16s RNA sequences using the 27F forward primer (5- AGAGTTTGATCMTGGCTCAG-3) and U1492R reverse primer (5- TACGGYTACCTTGTTAYGACTT-3) (Inqaba Biotec East Africa Ltd., Kampala, Uganda) according to the manufacturer’s instructions. PCR was performed using an Applied Biosystems SimpliAmp Thermocycler (Thermo Fisher Scientific, Waltham) under the following conditions: initial denaturation at 98°C for 5 minutes, 35 cycles of denaturation at 98°C for 30 seconds, annealing at 55°C for 30 seconds, extension at 72°C for 1 minute, and elongation at 72°C for 10 minutes. Initial denaturation at 98°C for 5 minutes was performed to ensure complete denaturation of the DNA template, especially for complex or GC-rich regions. The denaturation step at 98°C for 30 seconds in each cycle was sufficient to separate the DNA strands, while avoiding excessive thermal damage. An annealing temperature of 55°C was selected to provide optimal binding of primers to the target sequences, balancing specificity and yield. An extension temperature of 72°C, held for 1 minute, allowed for efficient synthesis by Taq polymerase, which was optimal at this temperature. Final elongation at 72°C for 10 minutes ensured that all incomplete DNA strands were fully extended, providing complete products for downstream analysis. A total of 35 cycles were selected to amplify enough of the target DNA while minimizing nonspecific amplification or PCR artifacts.

### 2.10. Nanopore library preparation and sequencing

The amplified DNA was purified using beads (CLEAN NA, Waddinxveen, Netherlands) and barcoded using a Rapid Barcoding Kit (Oxford Nanopore Technologies, Oxford, United Kingdom). Further cleaning and quantification were performed using a Qubit SQK-RBK114.96. DNA (20 ng) was loaded into a Flongle Sequencing Expansion Kit FXP-FSE002 (Oxford Nanopore Technologies). The amount of DNA (20 ng) was selected based on recommendations by Oxford Nanopore to ensure sufficient input for generating high-quality sequencing libraries while minimizing reagents (Oxford Nanopore Technologies, 2022). Nanopore sequencing libraries were constructed using the generated amplicons and internal control DNA to ensure robust sequencing results and to minimize batch variability.^[29]^ Sequencing yielded 483,200, 790,000, and 365,000 reads from 87, 49, and 78 pores in the dementia, control, and MCI samples, respectively, using the MinION platform (Oxford Nanopore Technologies), according to the manufacturer’s protocol. The range in the number of reads obtained reflects the variations in sequencing efficiency across different samples, which can occur because of pore usage, sample quality, or loading concentration. A quality score threshold of 9 was applied over 24 hours to ensure that only high-confidence reads were included, thereby enhancing data reliability. A quality score threshold of 9 represents a balance between read accuracy and the retention of sufficient reads for analysis.^[30]^ Reads longer than 200 bases that passed the quality checks were retained to ensure that they contained sufficient sequence information for accurate alignment and taxonomic classification. These reads were aligned against the Silva 138 database using Minimap2 software, chosen for its speed and accuracy in mapping long reads for bacterial identification. Custom R scripts (version 4.2.2) cleaned the data and generated phyloseq objects for the analysis. The analysis included recording the total OTUs and performing various comparisons using the Phyloseq and Vegan packages in R. Differences in alpha and phylogenetic diversity between groups were tested, and analysis of variance (ANOVA) was used to identify factors associated with variations in microbial composition between groups.

### 2.11. Sequence data and statistical analyses

Alpha diversity was assessed using the Chao1 and Shannon indices to evaluate the species richness and evenness within each group.^[31]^ Group comparisons were conducted using the Kruskal–Walli test followed by Dunn test with Bonferroni correction.^[32]^ Beta diversity was evaluated using principal coordinate analysis (PCoA) based on Weighted UniFrac distances and Bray-Curtis dissimilarity, with significance testing performed using PERMANOVA. Canonical analysis of principal coordinates (CAP) was used to differentiate predefined phenotypic groups based on their microbiome profiles. The significance of group separation was evaluated using ANOVA-like permutation tests to ensure that the observed differences were statistically robust and relevant to the study objectives.^[33]^

Differential abundance analysis was conducted to identify significant bacterial taxa associated with age and BMI. This analysis utilized the DESeq2 package, employing negative binomial generalized linear models, Wald tests, and Benjamini–Hochberg adjustment to quantify taxa with notable abundance changes, represented by log_2_ fold changes.^[34]^ LEfSe further identified bacterial genera that differentiated between groups, with statistical significance determined using the Kruskal–Wallis test and Linear Discriminant Analysis (LDA) (score threshold > 2.0, *P* < .05). Spearman rank correlation was used to assess associations between microbial diversity and clinical variables, including cognitive performance, as measured by MoCA scores (*P* < .05).

All analyses and visualizations were performed using R software (version 4.0.2) and relevant packages including vegan, DESeq2, and ggplot2.

## 3. Results

### 3.1. Participant characteristics

The study included 104 participants divided into 3 groups: 77 (74%) with AD, 14 (13.5%) with MCI, and 13 (12.5%) with cognitively healthy controls. The median age was 76 years (IQR: 70–83) in the AD group, 76.5 years (IQR: 68–83) in the MCI group, and 70 years (IQR: 69–71) in the control group. Most participants were female, with 63 (81.8%) in the AD group, 11 (78.6%) in the MCI group, and 9 (69.2%) in the control group.

One participant with AD had diabetes mellitus. The prevalences of hypertension were 32 (41.6%), 8 (57.1%), and 7 (53.9%) in the AD, MCI, and control groups, respectively. Regarding medication, 14 (18.2%) AD participants were on antihypertensives, NSAIDs, or antidiabetics, compared to 3 (21.4%) MCI participants and 2 (15.4%) controls (Table 1).

**Table 1:** Demographic and clinical characteristics of participants.

Factor	Level	AD (n = 77)	MCI (n = 14)	Controls (n = 13)	*P*-value
Age (yr)	Median (IQR)	76 (70, 83)	76.5 (68, 83)	70 (69, 71)	.048
Gender	Females	63 (81.8%)	11 (78.6%)	9 (69.2%)	.574
	Males	14 (18.2%)	3 (21.4%)	4 (30.8%)	
BMI	Median (IQR)	28.0 (23.5, 34.3)	25.6 (21.6, 30.3)	32.3 (28.7, 38.4)	.174
Diabetes mellitus		1 (1.3%)	–	–	.838
Hypertension		32 (41.6)	8 (57.1%)	7 (53.9%)	.447
Concomitant medication		14 (18.2%)	3 (21.4%)	2 (15.4%)	.920

### 3.2. Alpha diversity comparisons across control, MCI, and AD groups

**Figure 1.**
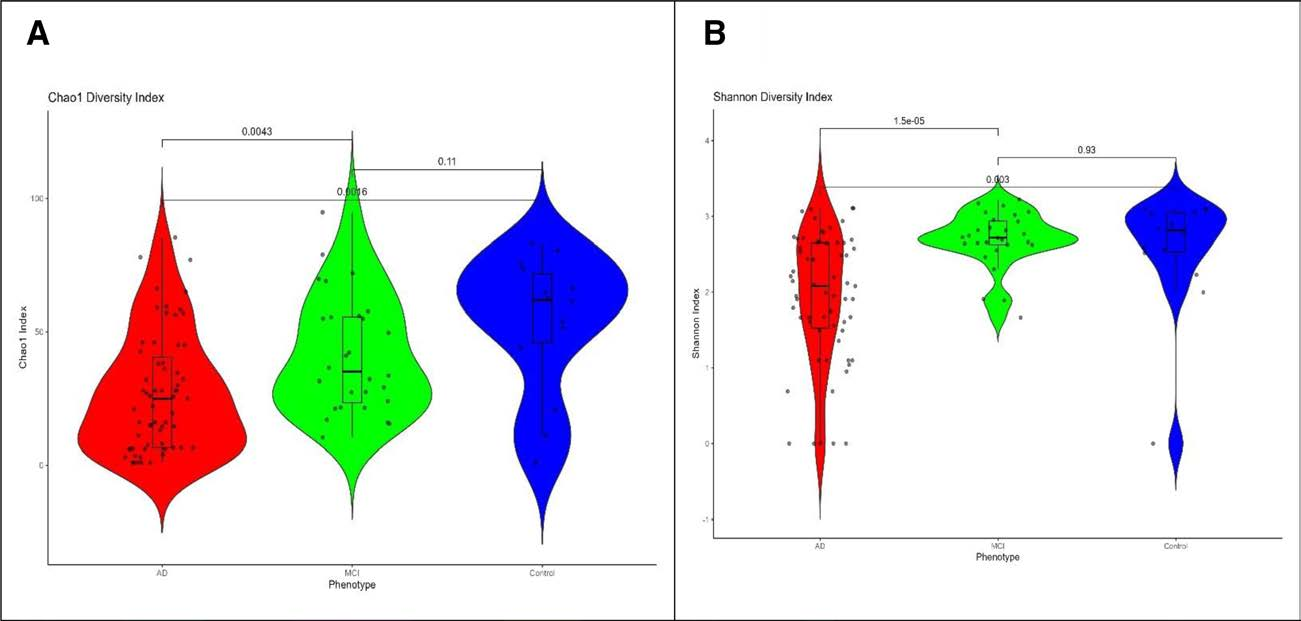
Alpha diversity indices in dementia (Alzheimer) group and there was a significantly lower richness in AD participants compared to MCIs by (a) Shannon index and (b) Chao1 index. Mid line shows median and error bars show interquartile range. AD = Alzheimer disease, MCIs = mild cognitive impairments.

The analysis revealed a clear pattern of decreasing microbial diversity associated with cognitive decline, as measured by both the Chao1 and Shannon diversity indices. The Chao1 diversity index for the control group displayed a broad distribution with the median value highlighted by the horizontal line in the violin plot (Fig. 1a). The control group exhibited higher Chao1 richness than the AD and MCI groups, indicating greater microbial diversity in the control group. The AD group had the lowest species richness, suggesting a potential loss in microbial diversity associated with disease progression. Statistical analysis confirmed that the differences between the control and AD groups were significant (*P* < .05), whereas the differences between the control and MCI groups approached statistical significance.

The Shannon diversity index, which assesses both species richness and evenness, reflects the overall biodiversity within each group (Fig. 1b). The Control group exhibited the highest Shannon Index, indicating a greater microbial diversity, whereas the AD group exhibited the lowest diversity. The MCI group fell between these groups, suggesting a decline in diversity as the cognitive impairment progressed. Statistical comparisons revealed significant differences in microbial diversity between the AD and control groups (*P* = 1.5e−05), and between the AD and MCI groups (*P* = .003). However, there was no significant difference between the MCI and Control groups (*P* = .93).

### 3.3. Beta diversity comparisons across control, MCI, and AD groups

**Figure 2.**
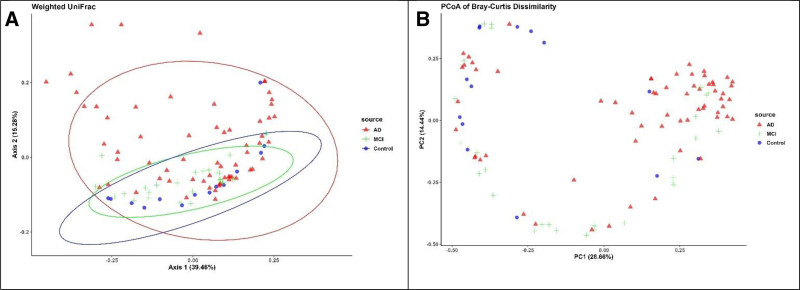
Comparison of beta diversity between the study groups. PC analysis plot showing weighted unifrac. Comparison of beta diversity between the study groups. PC analysis plot showing weighted UniFrac. The plot illustrates the beta diversity of microbial communities in stool samples from 3 groups: Control, MCI, and Dementia. The axes represent the first 2 principal coordinates, which explain 39.4% and 13.5% of the variance, respectively. Each point represents the microbial community of an individual subject, and the ellipses denote the 95% confidence intervals for each group. The analysis indicates distinct clustering of microbial communities among the groups, suggesting variations in the gut microbiota composition associated with cognitive status. (b) PCoA plot represents the beta diversity of gut microbial communities from 3 groups Beta-diversity by Bray-Curtis dissimilarity. The PCoA plot represents the beta diversity of gut microbial communities from 3 groups: Control, Dementia, and MCI. The axes represent the first 2 principal coordinates, which explain 28.66% and 14.64% of the variance, respectively. Each point corresponds to the microbial community of an individual subject, indicating the differences in microbial composition between the groups. The clear separation of points suggests distinct microbial profiles associated with cognitive status, with dementia patients showing the most divergent microbial communities. PC = principal cooridnate, PCoA = principal coordinate analysis, MCI = mild cognitive impairment.

The results revealed significant shifts in the microbial community structure associated with cognitive decline, as demonstrated by PCoA plots based on Weighted UniFrac distances and Bray-Curtis dissimilarity. The PCoA plot based on Weighted UniFrac distances (Fig. 2a) showed distinct clustering patterns among AD, MCI, and control groups. Axis 1, accounting for 39.46% of the variation, and Axis 2, explaining 16.28% of the variation, highlight a clear separation between the AD group (represented by red triangles) and control group (blue circles) along axis 1. This separation indicated a significant shift in the microbial composition associated with AD. The MCI group, depicted as green crosses, occupied an intermediate position between the AD and Control groups, suggesting that a gradient of microbial community changes correlated with cognitive decline. The 95% confidence ellipses for each group further emphasized these distinct microbial profiles, with a pronounced distinction between the AD and Control groups and some overlap between the MCI and Control groups.

Similarly, the PCoA plot based on Bray-Curtis dissimilarity (Fig. 2b) further demonstrated differences in microbial community composition across the AD, MCI, and Control groups. Here, the first principal coordinate (PC1) accounts for 28.66% of the variation, while the second principal coordinate (PC2) explains 14.44% of the variation. The plot shows a clear separation between the AD and control groups along PC1, indicating a significant shift in microbial composition linked to AD. The MCI group was more dispersed and partially overlapped with both AD and Control groups, reflecting a transitional microbial profile. This distribution pattern underscores the gradient of microbial community changes corresponding to cognitive decline, with the most pronounced differences observed between AD and Control groups.

### 3.4. CAP reveals distinct microbial community structures in control, AD, and MCI groups

**Figure 3.**
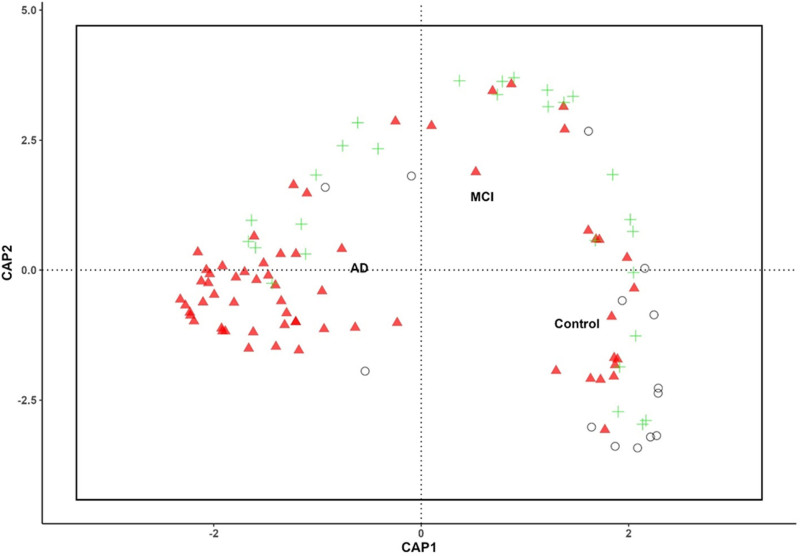
This CAP plot illustrates the ordination of gut microbiome samples based on their microbial community composition across 3 phenotypic groups: AD, MCI, and Control. The axes CAP1 and CAP2 represent the primary gradients of variation in microbial composition that distinguish these groups. The clustering of points suggests a distinction in microbial community composition between the AD, MCI, and Control groups, with AD samples clustering together, indicating a potentially unique microbiome profile associated with Alzheimer Disease. The MCI group exhibits a distribution that partially overlaps with both AD and Control groups, while Control samples are more distinct, indicating a different microbial composition compared to the AD and MCI groups. The separation along CAP1 and CAP2 highlights the underlying microbial differences contributing to the observed phenotypes. AD = Alzheimer disease, CAP = canonical analysis of principal coordinates, MCI = mild cognitive impairment.

The results revealed a clear differentiation in gut microbiome composition across AD, MCI, and control groups, as demonstrated by the CAP plot. The CAP1 and CAP2 axes capture the primary gradients of variation in microbial community composition (Fig. 3). The AD formed a distinct cluster on the left-hand side of the plot, indicating a unique microbial profile associated with AD. In contrast, the control group clustered to the right, suggesting markedly different microbial composition. The MCI group was positioned between the AD and Control groups, reflecting a transitional microbial profile bridging the 2. The separation along the CAP1 and CAP2 axes underscores the clear differences in gut microbiome composition among the 3 groups, with the AD group showing differences, followed by the MCI, and then Control groups.

### 3.5. Heatmap analysis of bacterial genera abundance in gut microbiome samples

**Figure 4.**
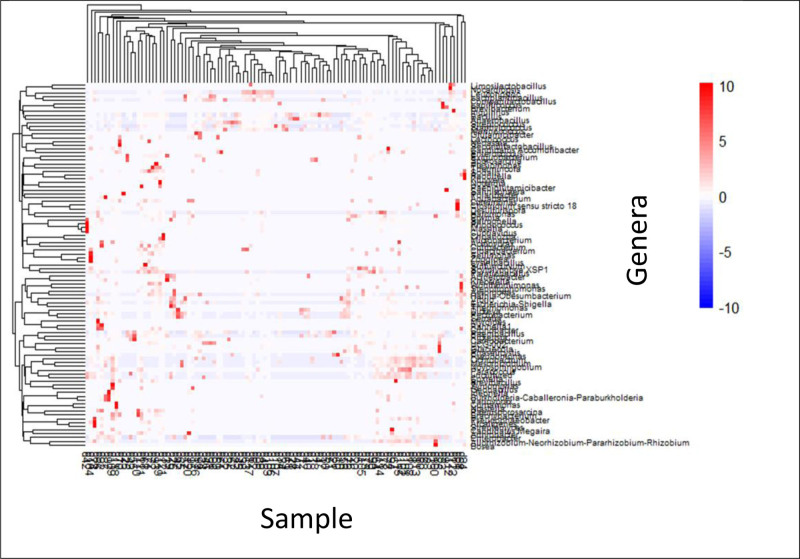
This heatmap illustrates the relative abundance of various bacterial genera across multiple gut microbiome samples, with samples arranged horizontally and bacterial genera listed vertically. The color gradient from blue to red indicates abundance levels, where blue represents lower abundance, white indicates medium abundance, and red shows higher abundance. The top and left dendrograms display hierarchical clustering, grouping samples with similar microbial compositions and clustering genera with similar abundance patterns across samples. These patterns and color variations highlight distinct microbial communities and emphasize genera that are abundant across different sample groups, making this visualization a valuable tool for comparing microbial diversity and composition in different conditions or phenotypes.

The results revealed significant patterns in the microbial community composition, as depicted by the heatmap showing the relative abundance of various bacterial genera across multiple gut microbiome samples (Fig. 4). In this visualization, the samples are arranged along the x-axis, whereas the bacterial genera are listed on the y-axis. The color gradient indicates abundance levels, with blue representing lower abundance, white indicating medium abundance, and red denoting higher abundance. Hierarchical clustering, depicted by dendrograms on both the top (for samples) and the left (for genera), highlighted groups of samples and genera with similar abundance patterns. Notably, distinct clusters of genera, such as *Lactobacillus*, *Bacteroides*, and *Clostridium*, were more abundant in specific sample groups, suggesting that differences in microbial community composition may be related to disease status or other phenotypic characteristics. The presence of red patches within the blue or white rows emphasizes the genera that are particularly abundant in certain samples, potentially serving as key indicators for distinguishing between different conditions.

### 3.6. Comparative analysis of bacterial division abundance across control, AD, and MCI groups

**Figure 5.**
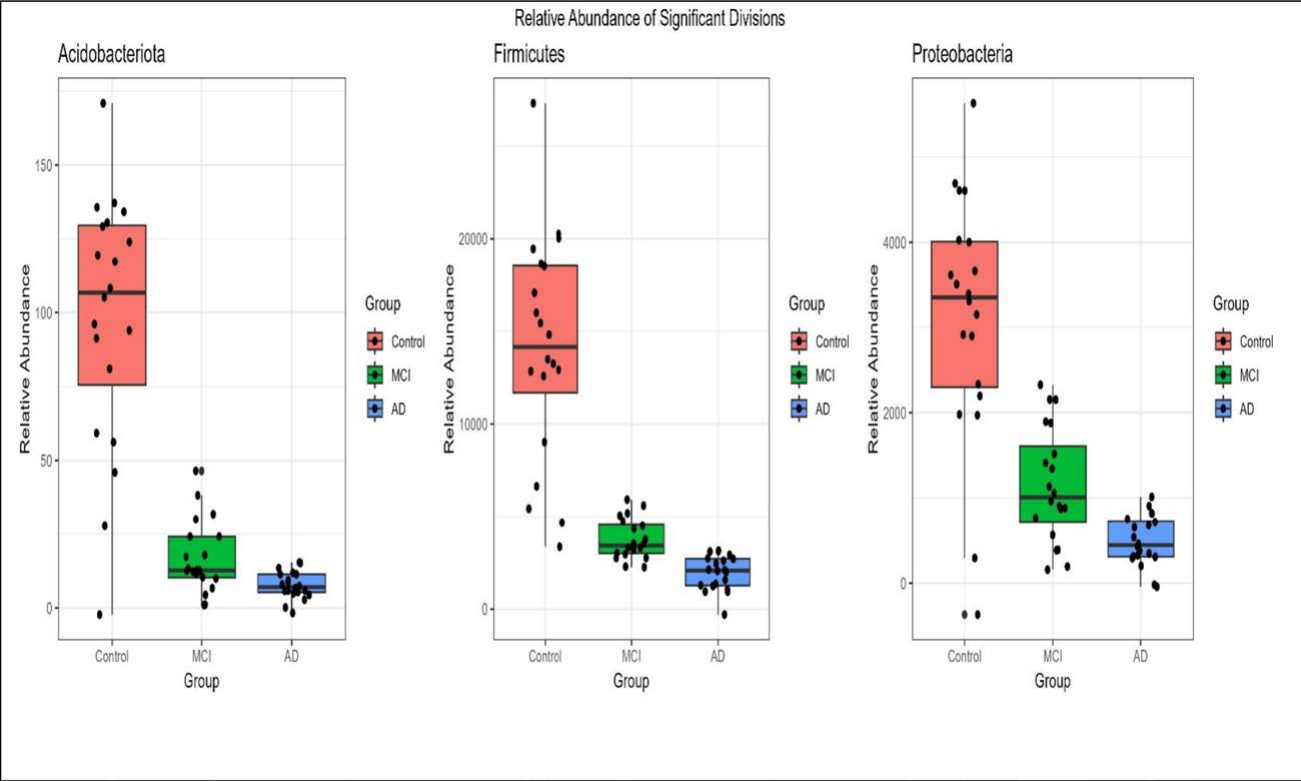
This figure presents the relative abundance of 3 significant bacterial divisions—Acidobacteriota, Firmicutes, and Proteobacteria—across 3 groups: Control, Dementia, and MCI. Each box plot displays the distribution of relative abundance within the groups, with the median marked by a horizontal line inside each box. The Control group is represented in blue, the Dementia group, and the MCI group. Whiskers indicate the range of the data, and individual dis points to highlight variability within each group. This comparison visualizes differences in microbial composition associated with dementia and MCI relative to the control group. MCI = mild cognitive impairment.

The results indicated significant differences in the relative abundance of key bacterial divisions across the control, dementia, and MCI groups. Box plots compare the abundances of *Acidobacteria*, *Firmicutes*, and *Proteobacteria* among these groups (Fig. 5). The relative abundance of *Acidobacteria* and *Firmicutes* was significantly higher in the control group than in the Dementia and MCI groups, with the control group displaying a broad range of abundance values. Conversely, both the Dementia and MCI groups showed a significantly lower abundance of these bacterial divisions, suggesting that a decline in these microbial populations is associated with cognitive impairment. A similar pattern was observed for *Proteobacteria*, where the Control group exhibited a significantly higher relative abundance than the Dementia and MCI groups did.

### 3.7. *Age-associated changes in gut microbiota: significant taxa with positive and negative log*_*2*_ *fold changes*

**Figure 6.**
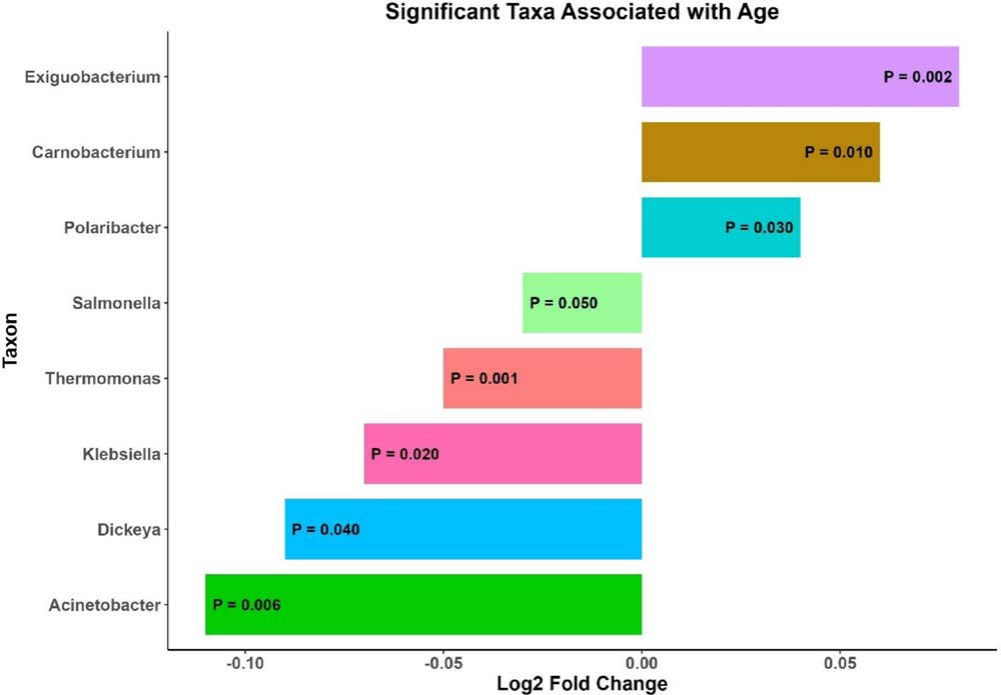
This bar plot illustrates the significant taxa associated with age, showing the log2 fold change of each taxon. The horizontal bars represent different taxa, with the length and direction of each bar indicating the magnitude and direction of the fold change associated with age. Taxa on the right side of the plot with positive log_2_ fold changes are more abundant with increasing age, while those on the left with negative log_2_ fold changes are less abundant with increasing age. The *P*-values displayed on each bar indicate the statistical significance of the association, with all highlighted taxa having *P ≤* .05, underscoring their significant relationship with age. The color of each bar represents different taxa, helping to distinguish them visually within the plot.

The analysis revealed significant shifts in bacterial taxa associated with aging, as indicated by their respective log_2_ fold changes (Fig. 6). Notably, certain taxa, such as *Exiguobacterium*, *Carnobacterium*, and *Polaribacter*, showed positive associations and their abundance increased with age. In contrast, several taxa, including *Acinetobacter*, *Dickeya*, *Klebsiella*, *Thermomonas*, and *Salmonella*, exhibited a significant decrease in abundance with age, as reflected by their negative log_2_ fold changes . The statistical significance of these associations (*P* < .05) underscores the robustness of our findings.

### 3.8. *Bacterial taxa significantly associated with BMI: positive log*_*2*_ *fold changes analysis*

**Figure 7.**
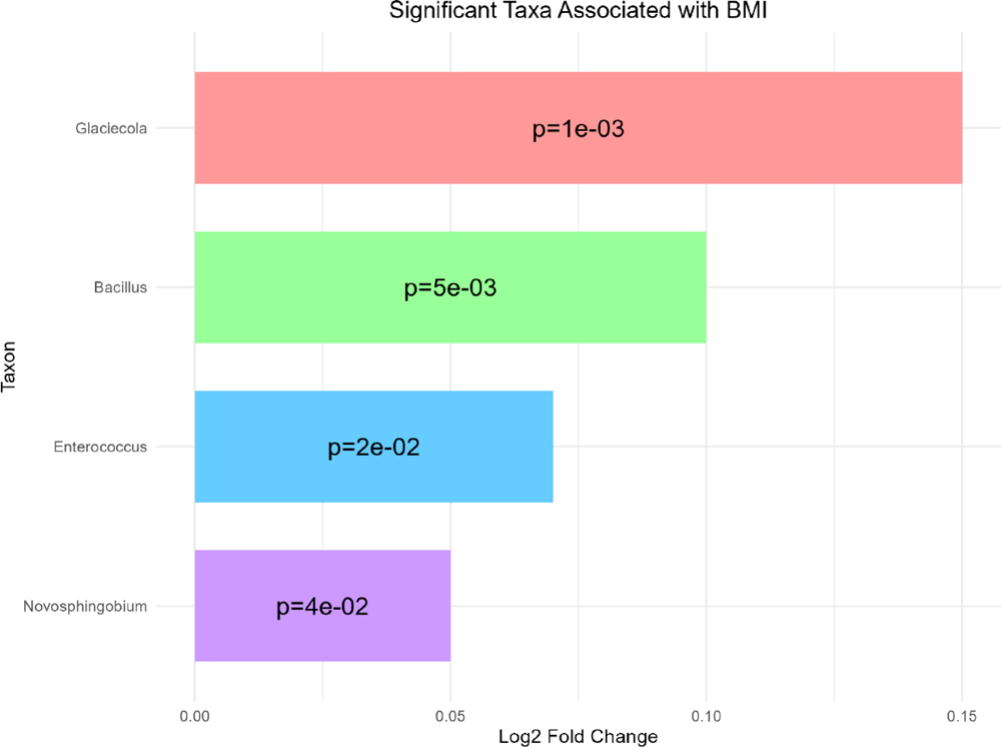
This bar plot displays the significant bacterial taxa associated with BMI, as measured by log2 fold change. Each horizontal bar represents a different taxon, with the length of the bar indicating the magnitude of the association between that taxon and BMI. The taxa are ordered by their effect size, with Glaciecola showing the highest positive association with BMI, followed by Bacillus, Enterococcus, and Novosphingobium. The colors of the bars help differentiate the taxa visually, providing a clear comparison of their relative contributions to BMI. This Figure highlights the taxa that are significantly more abundant as BMI increases, with all depicted taxa demonstrating a positive association with BMI. BMI = body mass index.

The results highlight significant associations between specific bacterial taxa and BMI, as depicted in the bar plot showing their log_2_ fold changes (Fig. 7). The taxa *Glaciecola*, *Bacillus*, *Enterococcus*, and *Novosphingobium* all exhibited positive associations with BMI, as indicated by their positive log_2_ fold changes values. Glaciecola demonstrated the strongest association with a *P*-value of 1e−03, followed by *Bacillus* with a *P*-value of 5e−03, *Enterococcus* with a *P*-value of 2e−02, and *Novosphingobium* with a *P*-value of 4e−02.

### 3.9. LEfSe analysis: LDA effect sizes for significant genera (MCI vs controls)

**Figure 8.**
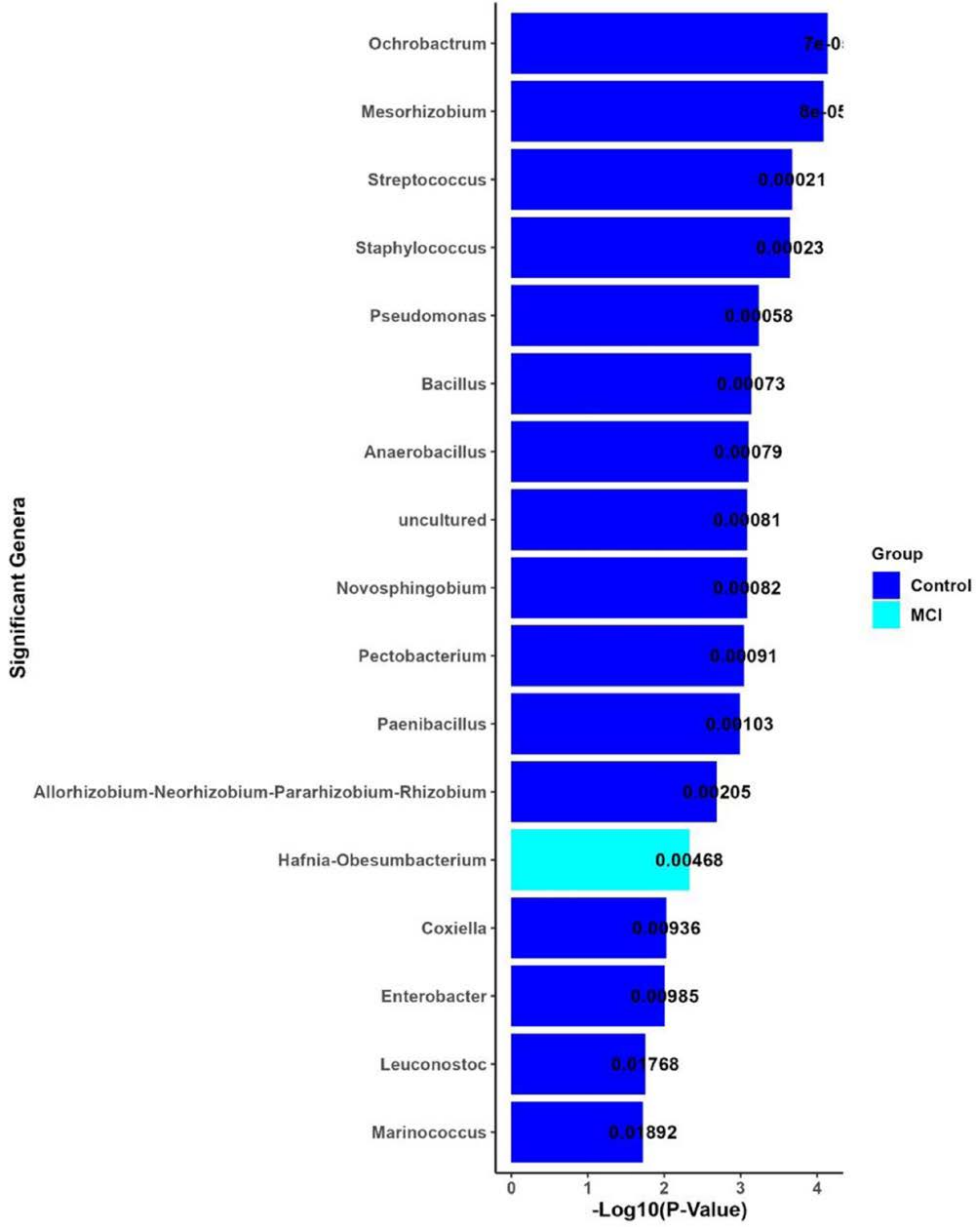
This bar plot shows the significant genera differentiating the control and MCI groups, based on the −log_10_(*P*-value). Each bar represents a genus, with the length of the bar indicating the statistical significance of the difference between the 2 groups. Genera are ordered by their significance, with Ochrobactrum and Mesorhizobium being the most significantly different between the groups. The bars are colored according to the group where the genus is more abundant: blue for the control group and cyan for the MCI group. The *P*-values displayed on the bars reflect the level of significance, with lower *P*-values (higher −log_10_(*P*-value)) indicating stronger evidence of a difference between the control and MCI groups. This figure highlights the specific genera that significantly contribute to the microbiome differences between individuals with MCI and those without cognitive impairment. MCI = mild cognitive impairment.

The results revealed significant differences in the gut microbiome composition between the control and MCI groups, as shown in the bar plot based on −log_10_(*P*-value) (Fig. 8). The bars represent various genera, with the length of each bar indicating the level of statistical significance; longer bars correspond to more significant differences between groups. The most significant genera, *Ochrobactrum* (*P* = 7e−07), *Mesorhizobium* (*P* = 8e−05), and *Streptococcus* (*P* = .00021), were more abundant in the control group (blue bars). In contrast, only one genus, *Hafnia-Obesumbacterium* (*P* = .00468), was more abundant in the MCI group (cyan bar). The P-values for each genus are annotated on the bars, with lower *P*-values reflecting stronger statistical significance.

### 3.10. LEfSe analysis: LDA effect sizes for significant genera (AD vs controls)

**Figure 9.**
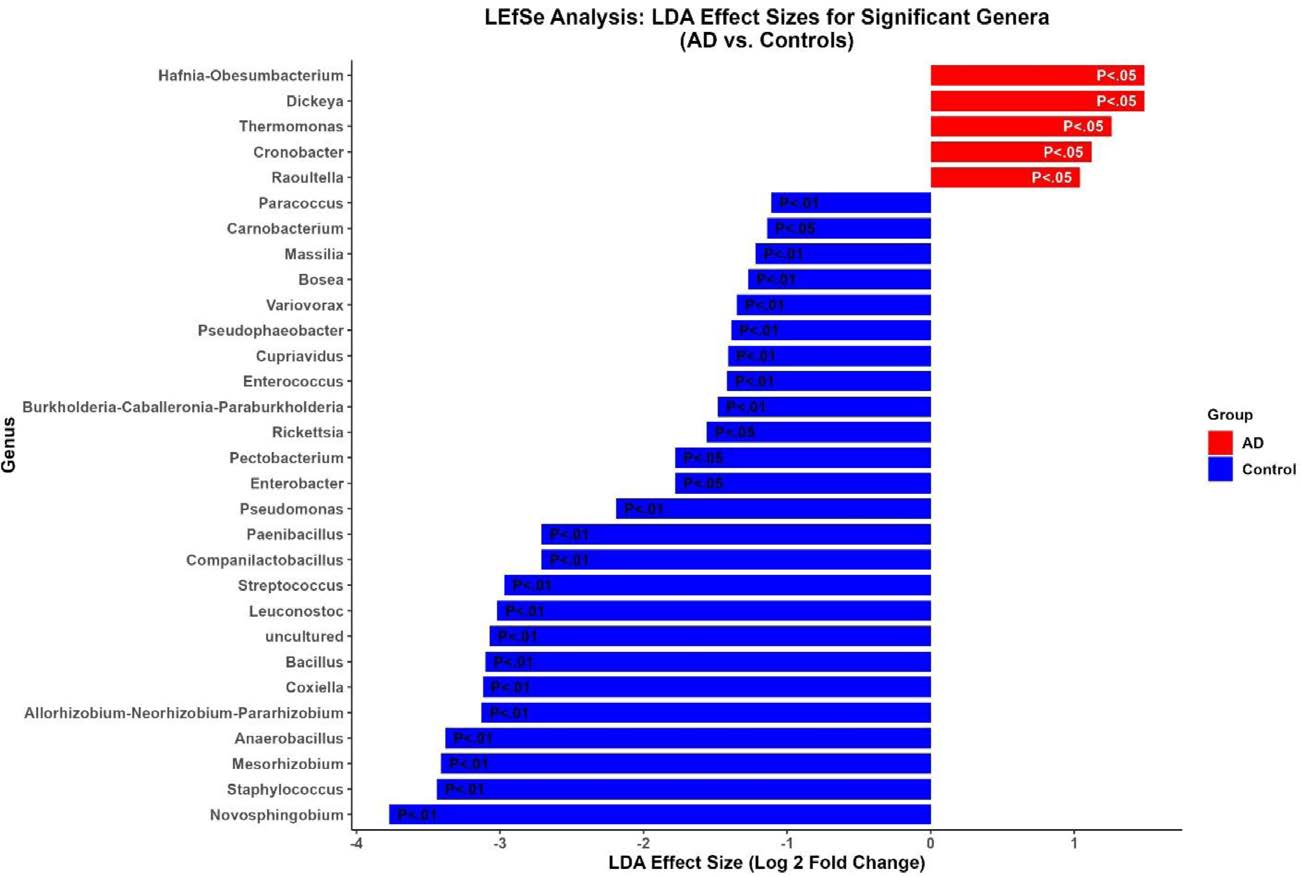
This figure presents the LDA effect sizes for genera that significantly differentiate between AD and control groups. The bar plot shows the LDA effect sizes (measured as log2 fold change) for each genus, with bars colored red for genera more abundant in the AD group and blue for those more abundant in the control group. Genera are listed on the y-axis, and the length of each bar reflects the magnitude of its effect size, indicating the strength of the association between that genus and either AD or control. *P*-values displayed on the bars indicate the statistical significance of these differences, with lower *P*-values denoting stronger evidence of differential abundance between the 2 groups. This figure highlights the microbial genera that are most distinctively associated with AD and those associated with healthy controls. AD = Alzheimer disease, LDA = linear discriminant analysis.

The results underscored the significant differences in gut microbiota composition between the AD and control groups, as shown in the LEfSe (LEfSe bar plot (Fig. 9)). Genera such as *Novosphingobium* (*P* < .01), *Staphylococcus* (*P* < .01), and *Mesorhizobium* (*P* < .01) were more abundant in the control group with negative LDA effect sizes, indicating their association with healthy individuals. Conversely, *Hafnia-Obesumbacterium* (*P* < .05), *Dickeya* (*P* < .05), *Thermomonas* (*P* < .05), and *Cronobacter* (*P* < .05) were more prevalent in the AD group with positive LDA effect sizes, highlighting their association with AD.

## 4. Discussion

This study provides valuable insights into the relationship between gut microbiome composition and cognitive decline, particularly in the context of AD and MCI. The primary finding of this study was a significant pattern of diminishing microbial diversity, which demonstrated a strong correlation with cognitive deterioration. The Chao1 and Shannon diversity indices revealed that individuals diagnosed with AD exhibited significantly lower microbial diversity than cognitively healthy controls. Additionally, the MCI group showed intermediate levels of diversity, suggesting that diversity of the gut microbiome may progressively decrease as cognitive impairment intensifies. This observation is consistent with a growing body of research suggesting that reduced gut microbiome diversity is implicated in the pathogenesis of neurodegenerative diseases.^[35,36]^ This observation aligns with a growing body of research, indicating that reduced gut microbiome diversity is implicated in the pathogenesis of neurodegenerative diseases.^[3]^ However, the literature has presented mixed findings. Some studies have reported no significant differences in microbial diversity across cognitive states, highlighting the complexity of the gut-brain axis and the need for further investigation. For instance, a 2022 study found no significant differences in alpha diversity between AD patients and healthy controls, suggesting that factors other than microbial diversity may play critical roles in disease progression.^[37,38]^

The significance of maintaining a diverse gut microbiome cannot be overstated as diversity is often indicative of a more resilient and stable microbial community that is better equipped to support host well-being. The reduction in diversity observed in patients with AD may reflect the loss of essential microbial functions that are vital for preserving neurological health, potentially through mechanisms such as inflammation modulation, neurotransmitter production, and gut barrier integrity.^[36]^ In contrast, some researchers have argued that specific changes in microbiome composition, rather than overall diversity, may be more critical in influencing neurodegeneration.^[39]^ The results of this study suggest that therapeutic strategies aimed at preserving or restoring gut microbiota diversity could be beneficial in reducing cognitive decline. This idea is supported by evidence from other studies that have demonstrated the potential of probiotic and dietary interventions to modulate the gut microbiome and improve cognitive outcomes.^[40]^

The findings from beta diversity analyses, including PCoA based on Weighted UniFrac distances and Bray-Curtis dissimilarity as well as CAP, indicated significant differences in microbial community composition among the AD, MCI, and Control groups. The distinct patterns observed, particularly the clear separation of the AD group from the control group, suggest that cognitive impairment is associated with specific changes in the gut microbiome. These changes in microbial composition may be triggered by various factors, including systemic inflammation, metabolic changes, and altered gut barrier function, all of which are influenced by the microbiome.^[41]^ However, some studies have shown that while compositional changes are evident, the functional consequences of these changes remain unclear, requiring more focused research on the mechanisms by which the microbiome influences cognitive decline.^[35]^

The study revealed a significant association between specific bacterial groups and factors such as age and BMI. For example, *Exiguobacterium*, *Carnobacterium*, and *Glaciecola* were positively associated with age and BMI, whereas *Acinetobacter and Klebsiella* were negatively associated. These findings suggest that as individuals grow older or experience an increase in BMI, notable changes occur in their gut microbiota composition, potentially affecting their susceptibility to cognitive decline.^[42]^ The relationship between BMI and specific gut microbiota raises intriguing questions regarding the interplay among metabolic health, obesity, and cognitive function. Considering the role of gut microbiome in the regulation of metabolic processes, obesity-related dysbiosis may contribute to or exacerbate cognitive decline. This perspective is supported by research linking metabolic syndrome with an increased risk of neurodegeneration through microbiome-mediated pathways.^[43]^ Nevertheless, other studies have suggested that the impact of BMI on cognitive function may be indirect and modulated by additional factors such as inflammation and insulin resistance, warranting further exploration.^[44]^

LEfSe analysis identified specific genera that were significantly associated with cognitive status, thus providing potential biomarkers for AD and MCI. For example, *Novosphingobium*, *Staphylococcus*, and *Mesorhizobium* were more prevalent in the control group, whereas *Hafnia-Obesum bacterium*, *Dickeya*, and *Thermomonas* spp. were associated with AD. These genera can serve as important indicators of disease progression or as targets for therapeutic intervention.^[35]^ The identification of such microbial biomarkers is particularly promising for the development of noninvasive diagnostic tools.^[45]^ Fecal microbiome analysis could potentially complement existing clinical assessments, provide a more comprehensive picture of a patient’s health status, and help predict the onset or progression of cognitive decline. Future studies should focus on validating these biomarkers in larger and more diverse cohorts and exploring the underlying mechanisms by which these microbes influence cognitive function.

This study’s focus on an African cohort adds a unique perspective to the global understanding of the gut-brain axis in neurodegenerative diseases, as it underscores the importance of including diverse populations in research. The microbiome profiles observed in this study may differ from those reported in populations of European ancestry, owing to genetic, dietary, environmental, or cultural factors that shape the microbiome in distinct ways. The significant prevalence of dementia in sub-Saharan Africa coupled with the unique microbiome profiles observed in this study highlight the need for further research in diverse populations. Understanding the gut-brain axis in different genetic and environmental contexts could lead to the discovery of novel therapeutic targets and strategies that are more effective in specific populations, which may inform public health initiatives aimed at preventing cognitive decline through lifestyle interventions that promote a healthy gut microbiome.

## 5. Strengths of the study

1. Novel insights into the gut-brain axis: Our study provides new insights into the association between gut microbiome alterations and neurodegenerative diseases, particularly AD and MCI. This adds valuable data to the growing body of research on the gut-brain axis, an emerging area of interest in neurodegenerative research.
2. Diverse study population: Including participants from different cognitive stages (AD, MCI, and controls) strengthens the study by demonstrating progressive changes in the gut microbiome across the spectrum of cognitive decline. This enhances our understanding of how gut microbiota evolves as cognitive impairment intensifies.
3. Comprehensive analysis: Our study utilized multiple diversity indices (e.g., Shannon and Chao1) and taxonomic profiling to provide a detailed characterization of microbial composition changes. This comprehensive analysis helps to understand both alpha and beta diversities, which are crucial for evaluating gut microbial shifts in relation to cognitive health.
4. Potential clinical applications: By identifying significant microbial markers associated with AD and MCI, our study paves the way for potential noninvasive diagnostic tools and therapeutic interventions. This focus on clinical applicability strengthens the impact and translational value of the research.
5. Rigorous methodology: The samples were collected and handled using standardized and sterile techniques to minimize contamination, while sequencing was performed with high-quality controls. This rigour enhances the reliability and reproducibility of the findings.
6. Interdisciplinary approach: The study integrates knowledge from microbiology, neurology, and data science, making it an interdisciplinary endeavor. This holistic approach allows for a broader understanding of how gut health influences neurological outcomes and provides multiple perspectives on disease mechanisms.

## 6. Limitations

The findings of this study are subject to various confounding factors that influence the gut microbiome, including diet, socioeconomic status (SES), and healthcare access. Although measures were taken to control these variables, some residual confounding factors may remain. Dietary patterns were not comprehensively assessed, and variations in SES and healthcare access could have introduced a bias. Conducted within a Ugandan cohort, the study’s results may not be directly applicable to other populations due to differences in genetics, environmental exposures, cultural practices, and lifestyle factors. This geographical and cultural specificity limits the generalizability of our results to non-Ugandan populations. Methodologically, the study used 16S rRNA sequencing, which is effective for identifying bacterial taxa but is insufficient for detecting species-level differences or microbial functions. Future studies employing metagenomic sequencing could provide more detailed insights into the functional roles of the microbiome in various diseases.

## 7. Conclusion

Our study provides compelling evidence of significant changes in the composition of the gut microbiome associated with AD and MCI. From a physical standpoint, these findings suggest that alterations in the gut microbiome contribute to neurodegenerative processes through mechanisms, such as increased gut permeability, systemic inflammation, and oxidative stress. The interplay between microbial metabolites and physiological pathways of the host could exacerbate neuroinflammatory responses, which are known to play a key role in the progression of neurodegenerative diseases. These insights highlight not only the crucial role of the gut microbiota in neurodegenerative disorders but also the potential for targeted interventions that modulate the microbiome to influence disease outcomes.

### 7.1. Generalisability of the study results

The generalizability of this study is strengthened by its diverse sample from urban and rural Uganda, which reflects varied lifestyles and environments. By including a broad age range and using standardized diagnostic criteria (MoCA, ICD-11, and DSM-5), the findings may be applicable to similar low-income and middle-income settings. However, the results may have limited external validity in populations with different genetic backgrounds, diets, or environmental exposure. Additionally, the small sample size, especially for the control and MCI groups, could affect generalizability. Further studies across diverse regions and larger cohorts are needed to confirm these findings and to expand their applicability to other populations.

## Acknowledgments

We would like to express our sincere gratitude to Ms Sylvia Nalwanga for her invaluable support and assistance throughout the study. We also extend our deep appreciation to Dr Eric Katagirya for his expert guidance and insightful contributions, which greatly enriched this research.

## Author contributions

**Conceptualization:** Kamada Lwere, Haruna Muwonge, Mark Kaddumukasa.

**Data curation:** Kamada Lwere, Aisha Nazziwa.

**Formal analysis:** Kamada Lwere, Aisha Nazziwa, Ian Guyton Munabi.

**Funding acquisition:** Kamada Lwere, Mark Kaddumukasa.

**Investigation:** Kamada Lwere, Ian Guyton Munabi, William Buwembo.

**Methodology:** Kamada Lwere, William Buwembo, Rheem Nakimbugwe, Ian Guyton Munabi.

**Project administration:** Kamada Lwere.

**Resources:** Kamada Lwere, Mark Kaddumukasa.

**Supervision:** Haruna Muwonge, Hakim Sendagire, Noeline Nakasujja, Mark Kaddumukasa

**Validation:** Martha Sajatovic.

**Visualization:** Kamada Lwere, Haruna Muwonge.

**Writing – original draft:** Kamada Lwere, Mark Kaddumukasa.

**Writing – review & editing:** Kamada Lwere, Martha Sajatovic, Scott M. Williams, Joy Louise Gumukiriza-Onoria, Denis Buwembo, William Buwembo, Rita Nassanga, Ian Guyton Munabi, Noeline Nakasujja, Mark Kaddumukasa.
